# Estimating the Prevalence of Knee Pain and the Association between Illness Perception Profiles and Self-Management Strategies in the Frederiksberg Cohort of Elderly Individuals with Knee Pain: A Cross-Sectional Study

**DOI:** 10.3390/jcm10040668

**Published:** 2021-02-09

**Authors:** Elisabeth Ginnerup-Nielsen, Robin Christensen, Berit L Heitmann, Roy D. Altman, Lyn March, Anthony Woolf, Henning Bliddal, Marius Henriksen

**Affiliations:** 1 The Parker Institute, Bispebjerg and Frederiksberg Hospital, 2000 Frederiksberg, Denmark; elisabeth.marie.ginnerup.laebo@regionh.dk (E.G.-N.); robin.christensen@regionh.dk (R.C.); berit.lilienthal.heitmann@regionh.dk (B.L.H.); henning.bliddal@regionh.dk (H.B.); 2 Research Unit of Rheumatology, Department of Clinical Research, University of Southern Denmark, University Hospital, 5000 Odense, Denmark; 3 Department of Public Health, Section for General Practice, Copenhagen University, 1014 Copenhagen, Denmark; 4 Division of Rheumatology and Immunology, University of California, Los Angeles, CA 90095, USA; journals@royaltman.com; 5 Department of Rheumatology, Royal North Shore Hospital, Institute of Bone and Joint Research, University of Sydney, St Leonards, NSW 2065, Australia; lyn.march@sydney.edu.au; 6 Bone and Joint Research Group, Royal Cornwall Hospital, Truro TR1 3HD, UK; anthony.woolf@btopenworld.com; 7 Department of Physical and Occupational Therapy, Copenhagen University Hospital, Bispebjerg and Frederiksberg, 2400 Copenhagen, Denmark

**Keywords:** knee pain, knee osteoarthritis, early OA, illness perceptions, self-management strategies, cross-sectional study, survey

## Abstract

Knee pain is an early sign of later incident radiographic knee osteoarthritis (OA). However, the prevalence of knee pain in the general population is unknown. Additionally, it is unknown how people with knee pain choose to self-manage the condition and if the perception of the illness affects these choices. In this study, 9086 citizens between 60–69 years old in the municipality of Frederiksberg, Copenhagen, Denmark, were surveyed, of which 4292 responded. The prevalence of knee pain was estimated, and associations between illness perceptions (brief illness perception questionnaire [B-IPQ]), self-management strategies, and knee symptoms were assessed. The prevalence of knee pain was 21.4% of which 40.5% reported to use no self-management strategies (non-users). These non-users perceived their knee pain as less threatening and reported less severe symptoms than users of self-management strategies. Further, we found that a more positive illness perception was associated with less severe knee symptoms. In conclusion, among Danes aged 60–69 years, the knee pain prevalence is 21.4%, of which 40.5% use no treatment and perceive the condition as non-threatening. These non-users with knee pain represent a subpopulation being at increased risk of developing knee OA later in life, and there is a potential preventive gain in identifying these persons.

## 1. Introduction

The European prevalence of radiographically confirmed symptomatic knee osteoarthritis (OA) is around 4% [1], and the Global Burden of Disease 2010 study [1] ranked hip and knee osteoarthritis (OA) as one of the highest contributors of years lived with disability. With an ageing population, increasing body weight, and sedentary life style, all factors linked to risk of knee OA [2,3], society may head towards a potentially explosive development in knee OA. The diagnosis of knee OA relies much on radiological changes in the knee [4]; however, intermittent knee pain by itself is a strong predictor of incident radiological knee OA [5,6,7], and studies have found that people often experience pain and decreased functional level before any radiological changes are evident [1,8]. By the time people are diagnosed with radiographic knee OA, they often experience a greater impact on their quality of life than patients with other chronic conditions such as cancer and heart diseases [9].

When faced with a benign but potentially life changing diagnosis of a chronic disease (such as knee OA), people (in general) develop an organized pattern of perceptions about their condition and how it impacts their lives [10]. These illness perceptions vary between individuals and have been shown to affect physical function. For example, a cohort study of patients with hand, spine, knee, or hip OA showed that patients with negative illness perceptions had significantly worse functional outcomes over six years compared to those with more positive perceptions [11]. Illness perceptions may also affect the way the individual chooses to handle the disease and seek health care assistance. A population-based study of elderly patients found that a notion that nothing can be done to treat arthritis, heart disease, or difficulty sleeping was associated with decreased utilization of preventative health services and decreased likelihood of being affiliated with a general practitioner [12]. Another study found that patients who initially believed that their symptoms would have serious consequences for them had a higher health care use over two years [13].

Various self-management strategies are being recommended for knee OA, and ESCEO and OARSI guidelines have advocated that structured exercise and weight loss should be core interventions in the treatment of OA along with pharmacological treatments [14,15,16,17]. Recent reviews recommend controlling obesity as an important aspect in the minimization of arthritic pain syndromes [18] and advocate the use of physical therapy [19] and obesity counselling to improve patient outcomes [20]. Furthermore, complementary and alternative medicine (CAM), such as nutritional supplements, herbal medicine, and acupuncture, are more or less accepted by authorities and have, for decades, been used consistently by patients for different health purposes including chronic joint pain [21,22,23,24,25,26,27]. It is, however, unknown how illness perceptions associate with the use of self-management strategies among people with knee pain.

Accordingly, there were two aims of this study: The primary aim was to quantify the prevalence of individuals with self-reported knee pain and knee OA (according to the NICE self-reported framework) in a representative sample of elderly individuals. Secondarily, we wanted to look for associations between self-management strategies and illness perception, intensity of knee pain and other knee symptoms, and health-related quality of life among elderly individuals with knee pain. Finally, we wanted to explore if different illness perception profiles exist and how these profiles differ in terms of self-management strategies, intensity of knee pain, and other knee symptoms, and health related quality of life among elderly individuals with knee pain.

We hypothesized that elderly individuals with knee pain, who use self-management strategies for their knee pain, have a more negative illness perception and have more severe symptoms than individuals who do not use self-management strategies. Further, respondents with a predominantly negative perception of their knee pain were hypothesized to report higher pain levels and lower quality of life and to more frequently use self-management strategies than people with more positive perceptions of their knee pain.

## 2. Materials and Methods

### 2.1. Design and Period

We performed a cross-sectional study as part of an ongoing prospective cohort study [28]. Study findings are reported according to Strengthening the Reporting of Observational Studies in Epidemiology (STROBE) guidelines [29]. The prospective cohort study was pre-registered at clinicaltrials.gov (accessed on 20 December 2020) (NCT03472300) and reviewed by the local health research ethics committee who deemed the study exempt from approval (j.no. 17024697). Data collection for this cross-sectional study was initiated on 6 September 2018, and ended 21 October 2018. No formal power calculation was performed due to the exploratory design, where we wanted to invite all the elderly citizens between 60 and 69 years of age. Thus, we anticipated a response rate of around 40 percent to include approximately 3500 participants to have approximately 1000 reporting knee pain [28].

Data was collected through a survey (“Frederiksbergundersøgelsen”) sent to all citizens aged between 60 and 69 living in Frederiksberg Municipality, in Copenhagen, Denmark. The survey was sent through the public “Digital Post” system (electronic mailbox for letters from Danish authorities) administered by the platform “e-Boks” [30], linked to the individual’s Personal Identification number—a national identification number, which is part of the personal information stored in the Civil Registration System. In the e-letter, an invitation to participate in the study was provided along with a link to an online survey that was managed using REDCap electronic data capture tools hosted at the capital region of Denmark. REDCap (Research Electronic Data Capture) is a secure, web-based software platform designed to support data capture for research studies, providing (1) an intuitive interface for validated data capture, (2) audit trails for tracking data manipulation and export procedures, (3) automated export procedures for seamless data downloads to common statistical packages, and (4) procedures for data integration and interoperability with external sources. (REDCap) [31,32]. The letter included information about the study as well as information about the rights to withdraw from the study at any time. Furthermore, the importance of responding to the questionnaire regardless of whether knee pain was present or not was emphasized.

### 2.2. Participants

Denmark is a country with a solid base for electronic communication. Most of the citizens have daily access to the internet, and Danes have a strong history of compliance with surveys. Frederiksberg Municipality was chosen for this study, being a very stable community with inhabitants rarely relocating. Inclusion criteria for the parent cohort study consisted of being between 60–69 years, living in the Municipality of Frederiksberg, being able to read and understand Danish, and having access to Digital Post. The age of 60–69 was chosen, as the incidence of knee OA increases with age, and an increasing prevalence of knee pain related disablement is encountered [1,33]. In this study, any knee pain during the last month when either sitting still or moving was an inclusion criterion as well. We had no formalized exclusion criteria.

### 2.3. Variables and Outcome Measures

The full survey description is available in the published protocol [28]. The questionnaires were adaptive based on how each respondent answered. It included a maximum of 189 questions for people reporting knee pain based on the initial triage question: “Have you experienced any pain from your knee/knees during the last month (both at work and rest)?”

The rest of the questions concerned use of conventional products and treatments and complementary and alternative medicines (CAMs) for knee pain/other reasons than knee pain, or general health. It furthermore concerned earlier knee injuries/surgeries, illness perceptions (related to knee pain), health related quality of life, musculoskeletal health, fitness and physical function, health beliefs, and attitudes concerning use of CAMs and physical activity, lifestyle, and demographics. The duration of knee pain was assessed based on predefined answers (0–6 months, 6–12 months, 1–2 years, 2–5 years, 5–10 years, and more than 10 years).

Furthermore, the following questionnaires were used:

#### 2.3.1. The Brief Illness Perception Questionnaire

We used the Danish version of The Brief Illness Perception Questionnaire (B-IPQ). B-IPQ is a generic nine-item questionnaire developed to rapidly assess the cognitive and emotional representations in a variety of illnesses. B-IPQ is a short version of the 84-item revised illness perception questionnaire (IPQ-R) [34].

The first eight items are scored on a 1–10 numeric rating scale with descriptors (none or extreme) at either end with 1 being no perceived threat in items 1, 2, 5, 6, and 8 (e.g., no symptoms/no consequences/no concern) and highest perceived threat (e.g., no illness control/no effect of treatment/no illness understanding) in items 3, 4, and 7 [35].

B-IPQ assesses perceptions on the following five dimensions: Identity, Cause, Timeline, Consequences, and Cure-Control. [36]. Five of the items assess cognitive illness representations: consequences (Item 1: “How much does your illness affect your life?”), timeline (Item 2: “How long do you think your illness will continue?”), personal control (Item 3: “How much control do you feel you have over your illness?”), treatment control (Item 4: “How much do you think your treatment can help your illness?”), and identity (Item 5: “How much do you experience symptoms from your illness?”). Two of the items assess emotional representations: concern (Item 6: “How concerned are you about your illness?”) and emotions (Item 8: “How much does you illness affect you emotionally? e.g., does it make you angry, scared, upset or depressed?”). One item assesses illness comprehensibility (Item 7: “How well do you feel you understand your illness?”). Item 9 is a free text field in which the respondent can formulate their beliefs about their condition (cause). We decided not to use this field in our study, as the data were inconsistent.

In all item questions, we replaced the word “illness” with “knee pain” as recommended when applying the B-IPQ to specific conditions [36]. Given that our respondents are not necessarily receiving any treatments and would therefore not be able to give a reasonable answer, the wording in item 4 was changed from “how much do you think your treatment can help your knee pain?” to “how much do you think treatment can help your knee pain?”. This change in wording of the item can affect the generalizability of the question to some extent, as the revised question assesses treatment expectations rather than control among participants not using treatments for knee pain.

The B-IPQ scores have shown good test–retest reliability and adequate concurrent, discriminative, and predictive validity amongst patient samples with musculoskeletal disorders and other chronic disorders [36,37]. Based on other studies [38,39,40] and the fact that each item represents one component (of many) found to underlie the cognitive representation of illness [41], we explored each B-IPQ item score and not the overall score.

#### 2.3.2. Knee Injury and Osteoarthritis Outcome Score

The Knee Injury and Osteoarthritis Outcome Score (KOOS) is developed as an instrument to assess the patient’s opinion about their knee and associated problems. It is patient reported and can be used to assess groups and to monitor individuals. The KOOS consists of 42 items covering five domains, namely, Pain (nine items), Symptoms (seven items), Activities of Daily Living (ADL) (17 items), Sports and Recreation (five items), and knee related QoL (four items). The KOOS adopts a five-point Likert scale scoring system (ranging from 0 (least severe) to 4 (most severe) [42].

A normalized score is calculated for each domain with 100 indicating no symptoms and functional impairment and 0 indicating extreme symptoms and functional impairment. KOOS has been validated for short- and long-term follow-up studies of knee injury [43] and is considered reliable and responsive for assessment of knee complaints in a comparative review of knee-specific outcome [44]. Minimal clinically important differences (MCIDs) for the KOOS is suggested to be 10–17 points [45].

#### 2.3.3. Current Knee Pain and Self-Reported Knee Osteoarthritis

Current knee pain, defined as “the level of pain in your knee today” was assessed with a 100 mm visual analogue scale (VAS) with anchors 0 = “no pain” and 100 = “worst imaginable pain”.

Self-reported knee OA was assessed corresponding to the definition made by The National institute for Health and Care Excellence (NICE) in 2014 [46]:

- Age over 45
- Activity related knee pain and
- Morning joint-related stiffness that lasts no longer than 30 min

#### 2.3.4. Health Related Quality of Life

To assess health related quality of life, we applied the EuroQoL five dimensions (EQ-5D) questionnaire. EQ-5D is a standardized measure of health status that provides a simple, generic measure of health. It is applicable to a wide range of health conditions and is ideally suited for use in surveys [47]. The EQ-5D consists of a descriptive system comprising five dimensions: mobility, self-care, usual activities, pain/discomfort, and anxiety/depression. Standardized answer options are given (three Likert boxes) and each question is assigned a score from 1 to 3. From the responses, an EQ-5D index is calculated ranging from −0.624 (worst) to 1.000 (best) [48]. EQ-5D is simple, taking only a few minutes to complete. A Danish version of the EQ-5D is available, and a Danish valuation set for reference is available [49].

### 2.4. Self-Management Strategies

We used the term self-management strategy as an overall definition of different treatment types (CAMs, conventional products, and conventional treatments) where the patient has an active role in choosing and administering the treatment.

### 2.5. Use of CAMs

We defined complementary and alternative medicines as either “alternative treatments”, “dietary supplements”, or “vitamins/minerals” taken specifically for knee pain.

- Vitamins and minerals were defined as any supplement containing only vitamins or minerals, taken by the respondent in order to relieve knee pain or promote health. Seventeen predefined choices for the use of vitamins or minerals were given (Supplementary file 1).
- Dietary supplements/herbal medicines (in this manuscript referred to as “dietary supplements”) were defined as any supplement not being a vitamin/mineral. Twenty-five predefined choices for the use of dietary supplements were given including fish oil, rosehip, ginger, glucosamine, probiotics, and medical cannabinoids (Supplementary file 1).
- Non-medical treatments were defined as an (active) treatment (normally) not being delivered by a medical doctor or another authorized health professional. Sixteen predefined choices for alternative treatments were provided, including: acupuncture, acupressure, cranio-sacral therapy, hypnosis, and kinesiology (Supplementary file 1).

In the survey, we asked if the respondents used any of these CAMs regularly specifically for their knee pain. Regular use of vitamins/minerals and dietary supplements was defined as “daily or almost daily use”, while regular use of non-medical treatments was defined as “use within last year”.

### 2.6. Use of Conventional Products and Treatments

“Conventional products” used specifically for knee pain were defined as over the counter (acetaminophen, codeine, NSAID) and prescription pain medicines (NSAID, opioids). Regular use of these drugs was defined as “daily or almost daily use”. We considered prescription pain medicine as part of self-management, as regular administration of this medication is, in contrast to surgery, very much relying on each patient. “Conventional treatments” were defined as physiotherapy, chiropractic, or weight loss. Use of these treatments was defined as use of physiotherapy/chiropractic “within last year” specifically for the knee pain and having “ever” tried to lose weight specifically to relieve knee pain.

We categorized participants reporting use of any type of treatment for their knee pain as being “users” (of self-management strategies), while respondents not using any type of treatment were categorized as “non-users”. In this process, we chose to combine conventional, proven effective treatments and CAMs as being all “self-management strategies”, as our goal was to explore how people choose to manage their knee pain regardless of what is recommended by the authorities.

## 3. Statistical Analyses

Incidence rate (IR) and prevalence rate (PR) are the two most important measures to assess the disease risk and occurrence in epidemiological studies like the Frederiksberg Cohort Study. The incidence rate takes the number of newly identified self-reported knee pain cases divided by people at risk in a defined period of time, while the definition of prevalence is comparable to the proportion of individuals with self-reported knee pain in the population at baseline. These two measures serve different purposes for the Frederiksberg Cohort Study. The PR shows how widespread the condition is, and thus provides us with information on the burden of disease in comparison to the estimated global burden of disease [50]. The calculation of the prevalence of knee pain and knee OA in the Frederiksberg Cohort study is presented in Supplementary File 2.

In order to evaluate the “true prevalence” of self-reported knee pain in this group of elderly citizens, a series of analyses were done based on what we considered the intention-to-survey population. Nonresponse in sample surveys was handled by replacing each missing value with multiple imputations [8,51]: multiple imputation (MI) was used to account for participants who were invited to join the survey but did not report the primary outcome: self-reported knee pain (yes/no). Five imputations as well as the original dataset were performed, and results from these six datasets were combined using Rubin’s Rules [51]. Based on these new “complete datasets, with no missing data” we applied standard complete-data methods to analyze the multiply-imputed sets using frequentist ideas (results combined using Rubin’s Rules [51], as well as applying simple bootstrap resampling techniques to estimate the empirical range from minimum to maximum for the observed sample [52]).

The exploration of the associations between type and extent of self-management strategies, illness perception (B-IPQ), intensity of knee pain and other symptoms, and health related quality of life was done in several steps. We initially compared the two groups we had created (“users” and “non-users”) on demographics and questionnaire data. As data did not follow a normal distribution, Kruskal–Wallis’ non-parametric tests for independent samples were conducted to compare user/non-user groups and explore the association between the number of treatment types used and the B-IPQ, KOOS, Current VAS Pain, and EQ-5D.

To investigate if different illness perception profiles existed and how these profiles differed in terms of self-management strategies and health related outcomes measures, we performed a cluster analysis (CA) based on the B-IPQ-item scores and related these clusters to the distribution of users/non-users, knee pain intensity (KOOS pain and current VAS), other symptoms (KOOS), and health-related quality of life (EQ-5D). Based on Frostholm et al. [53] and recommendations in the literature [54], we applied a two-step procedure: (1) a hierarchical analysis (Ward’s method) using squared Euclidean distance to determine the optimal number of clusters based on a reformed agglomeration schedule. An inconsistent decrease in the coefficient score is used to indicate that the clusters at this point are distinct and therefore the cluster process should be stopped one step earlier [53]. We then used the centroids from the hierarchical CA as a starting point in a *K*-means CA with a predefined number of clusters to validate the results from the hierarchical analysis.

Differences between clusters in age, knee pain duration, BMI, health related outcomes measures, and pain levels were assessed using Kruskal–Wallis test for non-parametric independent samples. The distributions of males/females and users between the clusters were assessed using Chi^2^ test. As the B-IPQ only contains one type of scale (scores 1–10), no standardization was required.

All analyses were performed in SPSS (version 3.3). All P-values and 95% confidence intervals were two-sided. We did not apply explicit adjustments for multiplicity; rather, we interpreted the findings from the multiple tests performed, considering the serious risk of making a false discovery (i.e., a false-positive inference). The statistical tests were reported with P-values for standard hypothesis tests, and any claim of statistical significance was only intended for exploratory purposes (with a statistical α level of 0.05).

## 4. Results

Among a total of 9086 citizens in the Frederiksberg Community aged between 60–69 years, 882 did not have access to e-Boks. Thus, 8204 were invited to respond to the survey. At the end of the six-week data collection period, 4292 (52.3%) had initiated the questionnaire. Among these, 1758 (40.9%) reported knee pain and 570 (13.3%) reported self-reported knee OA corresponding to NICE self-reported framework.

From our bootstrap resampling technique, we have an empirical interval around the proportion having self-reported knee pain ranging from 19.9% to 22.7%. From this sample, it is fair to assume that the prevalence of knee OA is between 6.4% and 7.4% in this group of individuals in the age 60 to 69 years of age (Supplementary File 2). However, since this is probably a low-level guestimate, we also applied a conservative multiple imputation technique replacing the missing data (i.e., based on a tipping point analysis strategy); from these repeated datasets (combined using Rubin’s rule) a conservative estimate on individuals with self-reported knee pain could be as high as 54.2% (rather than 21.4%). As a consequence, the prevalence of knee OA in this sample might be as high as 17.6%.

**Figure 1:**
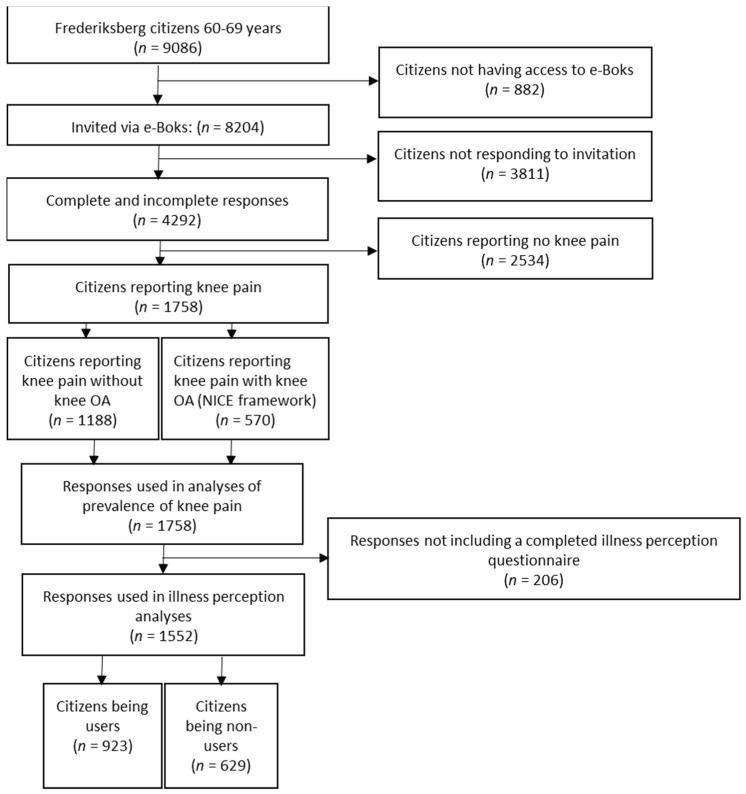
Flow chart response flow.

The analyses on illness perceptions, functional level, current VAS knee pain, and quality of life are based on respondents who reported knee pain, and had responded to all B-IPQ items and at least one of the following measures: KOOS, current VAS, or EQ-5D (*n* = 1552, 34.9%) (Flowchart Figure 1).

Among these 1552 respondents reporting knee pain, 64% were women. The duration of knee pain was evenly distributed across less than one year to more than 10 years.

A summary of the demographics and questionnaire data is presented in Table 1.

### 4.1. Self-Management Strategies for Knee Pain

Of the 1552 respondents, 923 (59.5%) reported use of any kind of treatment or supplement specifically for their knee pain. Hence 59.5% of the respondents were categorized as “users” (of self-management strategies), the rest (*n* = 629; 40.5%) as “non-users”. Of the 923 users, 398 (43%) reported to use only one type of treatment, while only nine respondents (1%) reported use of more than five treatment types. Of the 923 users, 374 (40.5%) reported self-reported knee OA.

The proportions of women among the users and non-users were 66.3% and 60.6%, respectively, with statistically significant difference (*p* = 0.021).

### 4.2. Brief Illness Perception Scores in Users and Non-Users

We found differences between users and non-user on all B-IPQ items except for items 7 and 8. On items 1, 2, 4, 5, and 6, users scored higher than non-users (Table 1). On item 3, non-users scored higher than users (Table 1). Altogether, this suggests that non-users perceived their illness as less threatening than users.

The highest average scores were seen on item 2 (Timeline) “How long do you think your knee pain will continue?” with a median score of 8 (IQR 5-10) for non-users and 10 (IQR 6-10) for users. Accordingly, both groups expected their pain to continue for very long with users considering the condition to last longer than non-users.

The lowest average scores concerned the B-IPQ item 8 (Emotional representation)—“How much does your knee pain affect you emotionally?” with a median of 2 (IQR 1-2) for non-users and 2 (IQR 1-5) for users, indicating that both users and non-users were only a little emotionally affected by their knee pain. (Table 1).

On item 3: “How much control do you feel you have over your knee pain?”, non-users had higher median scores than users (difference: 1 (95% CI: 0.55 to 1.45)), suggesting that they felt they had more control over their pain than users. On item 4, “How much do you think treatment can help your knee pain?”, users had a higher median score than non-users (difference: 2 (95% CI: 1.57 to 2.43)) suggesting that they found treatment more likely to help them than non-users did (Table 1).

### 4.3. Health-Related Outcome Measures

Non-users scored significantly lower (less symptoms) than users on all KOOS subscales with the largest differences observed in the KOOS Sports and Recreation and KOOS QOL subscales (median difference respectively: 25 (95% CI: 21.0 to 28.34) and 12.6 (95% CI: 11 to14.2)) and the smallest difference on the KOOS symptoms subscale (median difference: 7.2 (95% CI: 5.9 to 8.5)). Likewise, EQ-5D scores were also significantly higher among non-users than users (median difference: 0.048 (95% CI: 0.039 to 0.057)), while the median Current VAS Pain was 13 points lower (95% CI: −15 to −11) among non-users compared to users (Table 1).

**Figure 2:**
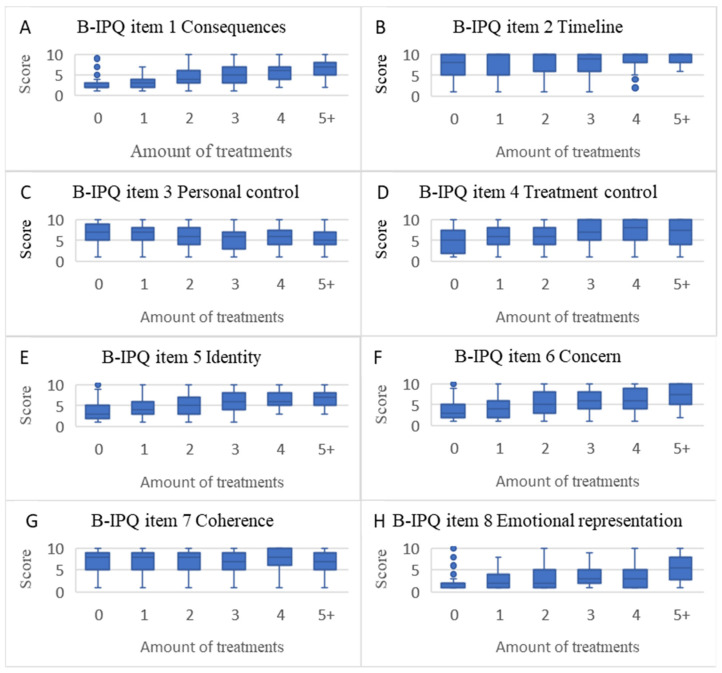
Boxplots of association between amount of treatments used and Brief Illness Perception Questionnaire scores (median, upper, and lower quartiles and outliers). (**A**): B-IPQ item 1 consequences. (**B**): B-IPQ item 2 timeline (**C**): B-IPQ item 3 personal control. (**D**): B-IPQ item 4 treatment control. (**E**): B-IPQ item 5 identity. (**F**): B-IPQ item 6 concern. (**G**): B-IPQ item 7 coherence. (**H**): B-IPQ item 8 emotional representation.

**Figure 3:**
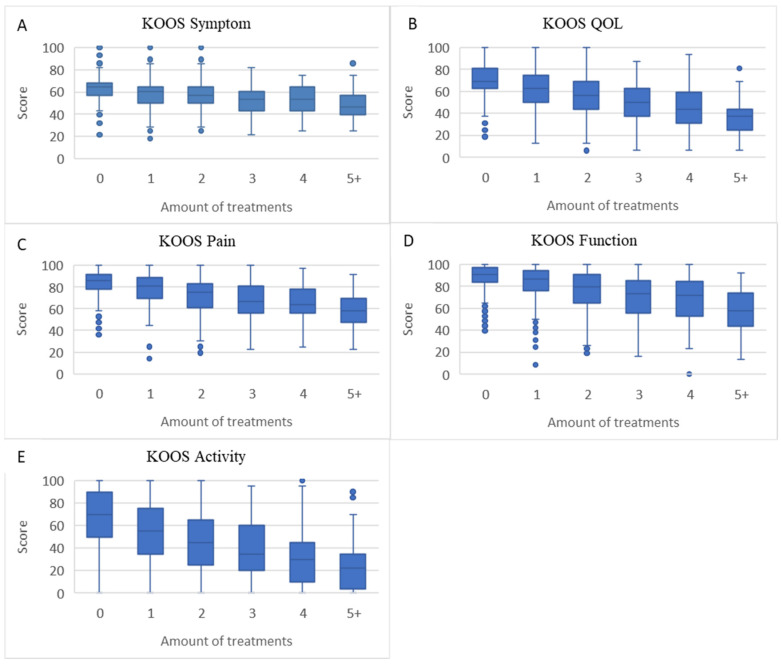
Boxplots of association between amount of treatments used and KOOS scores (median, upper, and lower quartiles and outliers). (**A**): KOOS Symptom, (**B**): KOOS QOL. (**C**): KOOS Pain. (**D**): KOOS Function, (**E**): KOOS Activity.

**Figure 4:**
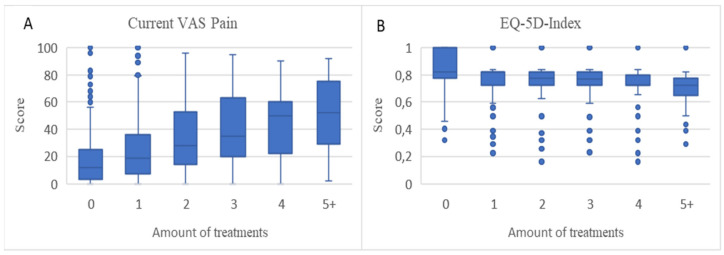
Boxplots of association between amount of treatments used and current VAS pain scores and EQ-5D scores (median, upper, and lower quartiles and outliers). (**A**): Current VAS Pain. (**B**): EQ-5D-Index.

There were statistically significant associations (*p* < 0.0001) between the number of treatments used for knee pain and B-IPQ scores (except for item 7) (Figure 2A–H), KOOS scores (Figure 3A–E), VAS pain scores (Figure 4A), and EQ-5D scores (Figure 4B), suggesting that with more treatments used the illness perception was more negative and the symptoms and quality of life were worse.

### 4.4. Cluster Analysis of Brief-IPQ Scores

Based on the change between coefficients in the agglomeration schedule from the hierarchical cluster analysis, we found a solution of two clusters to be optimal. In the next step, we used the centroids of the clusters in a K-means cluster analysis with a pre-set number of two clusters.

We found statistically significant differences between all B-IPQ item scores except item 7 as well as between all health-related outcome measures and Current VAS Pain in the two-cluster solution (Table 2).

The population in Cluster 1 had higher current VAS pain, higher BMI, and lower scores on EQ-5D and KOOS than cluster 2. Thus, cluster 1 was more affected on all health-related outcomes measures and perceived their pain as more “threatening” than cluster 2. Additionally, 481 (25.1%) of respondents in cluster 1 were characterized as non-users (Table 2).

Cluster 1 included 642 respondents. It was characterized by higher B-IPQ scores than cluster 2 on all items except for items 3 and 7 (Table 2). Based on the B-IPQ pattern, this cluster was named “concerned optimists”.

Cluster 2 included 910 respondents. Respondents in cluster 2 had lower B-IPQ scores than cluster 1 on all items except for items 3 and 7 (Table 2). Based on the B-IPQ pattern this cluster was named “unconcerned confident”.

Both clusters had similar scores on item 7 (coherence), suggesting that the two clusters had the same understanding of their knee pain (Table 2). The two clusters were similar with respect to age (median 64 in both clusters). The proportions of women in the two clusters were 64.8% in cluster 1 and 63.4% in cluster 2 (*p* = 0.57), while the proportions of self-reported knee OA were 50% in cluster 1 and 19.6% in cluster 2 (*p* < 0.0001).

## 5. Discussion

In this cross-sectional study, based on 4292 elderly individuals from the Frederiksberg Cohort, 1758 reported knee pain with 570 reporting symptoms corresponding to knee OA. The prevalence of knee pain and knee OA in the whole Frederiksberg Cohort (8204) was estimated to be, respectively, 21.4% and 6.9%, which is a little lower than found in other studies [1,33,55].

To our knowledge, this is the first study to document associations between illness perceptions and management patterns of knee pain in a large sample of individuals between 60 and 69 years. We found that users of self-management strategies for knee pain are characterized by more negative illness perceptions, worse symptoms, and lower health related quality of life than non-users. Further, we identified a group of individuals that, even though they reported having knee pain and being functionally affected did not use any self-management strategy.

When compared to populations with manifest radiological knee OA included in clinical studies, users reported slightly less pain and better functional level (KOOS), while part of the non-users had a significantly better functional level [44,56]. Knee pain levels among non-users were comparable to other studies of patients with early OA [6,57], suggesting that the non-users in this study are at increased risk of developing radiographic knee OA.

Even if functional levels were relatively high among respondents, it is evident that levels are still markedly lower than in an age-matched population without knee issues [58] and while non-users, not surprisingly, report less symptoms than users, it is striking that some non-users report significant symptoms, with 19.9% actually reporting symptoms corresponding to self-reported knee OA. On the other hand, the non-users’ median EQ-5D score is comparable with the Danish population norms [59], suggesting that their knee pain did not impact their overall quality of life.

Our results are similar to those found in a review of studies concerning the association between illness perception and different functional and psychological health outcomes measures among knee or hip arthroplasty populations [60], where higher scores on the B-IPQ items concerning consequences, identity, coherence, and emotional representation were predictive of worse knee function, pain interference with walking, and anxiety and depression. Another study of 1204 Irish citizens with chronic pain [61] found higher scores on timeline and concern items (items no. 2 and 6) to be predictive of pain-related disability.

We also found that illness perception, knee symptoms, and health related quality of life were associated with amount of treatments used. The more threatening respondents perceived their knee pain and the more physically impaired they were, the more treatments they reported to use. Similar results were found in a study by Hill et al. [39] concerning 2113 older people with musculoskeletal hand disorders, where participants who considered their disorder to have the most negative effects on their lives were more likely to consult a physician, take medication, or both.

On the other hand, a study by Bedson et al. [62] found that around 50% of individuals aged over 50 with disabling knee pain did not consult for it. Recent onset and severity of pain was associated with more use of general practice.

Together, these results indicate that experiencing knee pain is not necessarily considered a serious health threat to people, which could explain why many people do not consult general practice. As resent research [5,6,7] has shown intermittent knee pain to be a predictor of radiographic knee OA, it seems relevant to inform the general population about the potential gain of reacting with knee OA preventing measures to even mild knee pain. This would also be in line with earlier studies [63,64,65], emphasizing the effect of using population-based approaches to control determinants of incidence instead of individually targeted approaches to patients being at high risk or already having manifest disease.

Our cluster analysis revealed two clusters based on illness perception traits. Cluster 1 was characterized by worse illness perceptions, more pain, lower quality of life, and significantly worse health outcome measures than cluster 2. Cluster 2 had more positive illness perceptions but lower treatment control scores and better health outcome measures. Cluster 1 had a markedly higher proportion of users than cluster 2 (74.9% vs. 48.6%). The differences between clusters in especially KOOS subscales are highly clinically relevant and cannot only be explained by the distribution of users in the groups. The difference supports the view that illness perceptions and knee pain related functional level are closely related, and that a considerable part of the respondents use no self-management strategy despite being affected by their knee pain (25.1% in cluster 1).

Other studies have used cluster analyses based on illness perceptions to identify subgroups and relate these to management of a disease. Lowe et al. [66] examined the difference between use of unscheduled health care services in UK among three clusters based on the B-IPQ on patients with both chronic and non-chronic disorders, while Frostholm et al. [53] identified three clusters based on IPQ-R and related these to the use of primary care in Denmark. Both studies found that clusters where patients perceived their pain as having most consequences for their lives had more primary care visits. Riviera et al. [67] identified three clusters based on 187 patients with heart failure, chronic obstructive pulmonary disease (COPD), and chronic kidney disease. The cluster perceiving the illness to have few consequences and a non-fluctuating pattern had the fewest hospitalizations while both the cluster perceiving many consequences to their illness and the cluster perceiving a high disease control and understanding of their illness had many hospitalizations. These studies support our results of more negative perceptions being related to higher use of health care services, but also indicate that more negative perceptions of an illness might not be consequently related to use of more health-related services. As health services operate differently in each country, and as we did not measure hospitalizations, these results are not directly comparable to our results. Nevertheless, the results indicate that different illness perception profiles exist and are related to illness management.

This study has some limitations. First, as this is a cross-sectional study, we cannot conclude on the causality of the relationship between illness perception traits, management patterns, and health related outcome measures we have found. Our study population was sampled from a relatively wealthy area in Copenhagen, which may limit generalizability. Further, we defined “management of knee pain” as the use of different predefined treatment strategies based on recommendations and also the most common CAMs, which may not be the best way to describe an individual’s way of managing knee pain. However, we chose to include CAMs as a treatment for knee pain regardless of recommendations, as many people use them and believe in their effect, and as the effect of many types of CAM has not yet been investigated, we considered it relevant to include these.

As CAMs are often broadly defined depending on country or region [21,22,24,25,26,68,69], generalization related to CAMs in general should be done with caution.

The strengths of this study include a large sample size and a good response rate. Data was collected in an electronic survey questionnaire with branching, ensuring that all responses were presented and collected correctly. Another strength is that the main findings related to the association between illness perception and use (or non-use) of self-management strategies were documented using two analytical approaches.

## 6. Conclusions

In conclusion, we found the prevalence of knee pain in the Frederiksberg Cohort of elderly between 60–69 years to be 21.4%, while the prevalence of self-reported knee OA was 6.9%. We further found that elderly individuals with knee pain that use treatments and supplements as management strategies seem to be more concerned and have more severe symptoms than individuals that use no treatments or supplements. Additionally, we documented that worse illness perception was associated with higher degrees of knee symptoms, lower quality of life, and a tendency to use more treatments for knee pain. A large proportion of the elderly are not using any treatments even though they are affected by their knee pain, with 19.9% reporting knee symptoms corresponding to self-reported knee OA. These non-users with knee pain represent a subpopulation with an increased risk of developing radiographic knee OA, and there may be a potential preventive gain in identifying these persons that are not yet patients but report symptoms of early OA.

## Figures and Tables

**Table 1:** Characteristics of participants reporting knee pain and being users or non-users of self-management strategies.

	Users	Non-Users	Difference	*p* *
			Median (95% CI)	
N (%)	923 (59.5)	629 (40.5)	N/A	
**Demographics**				
Women, N (%)	612 (66.3)	381 (60.6)	N/A	0.021
age years, median (IQR)	64 (62–67)	64 (62–67)	N/A	0.35
BMI (kg/m^2^), median (IQR)	26.6 (23.6–30.5)	24.8 (22.6–27.9)	−1.8 (−2.27 to −1.33)	<0.0001
Knee OA ^¶^	374 (40.5)	125 (19.9)	N/A	<0.0001
**KOOS. 0–100 score (median, IQR)**				
KOOS symptoms	57.1 (50.0–64.3)	64.3 (57.1–67.9)	7.2 (5.9 to 8.5)	<0.0001
KOOS QOL	56.2 (43.8–68.8)	68.8 (62.5–81.2)	12.6 (11 to 14.2)	<0.0001
KOOS pain	75.0 (61.1–86.1)	86.1(77.8–91.7)	11.1 (9.7 to 12.5)	<0.0001
KOOS function	80.9 (66.2–91.2)	91.2 (83.8–97.1)	10.3 (8.8 to 11.8)	<0.0001
KOOS Sports and recreation	45 (25–70)	70.0 (50–90)	25.0 (21.7 to 28.3)	<0.0001
**EQ-5D Index (median, IQR)**	0.776 (0.723–0.824)	0.824 (0.776–1.000)	0.048 (0.039 to 0.057)	<0.0001
**Current VAS pain, 0–100 mm (median, IQR)**	25 (10.75–50)	12 (3–25)	−13 (−15 to −11)	<0.0001
**Brief-IPQ, 1–10 score (median, IQR)**				
consequences B-IPQ 1	4.0 (2–6)	2.0 (2–3)	−2.0 (−2.3 to −1.7)	<0.0001
timeline B-IPQ 2	10.0 (6–10)	8.0 (5–10)	−2.0 (−3.1 to −0.9)	<0.0001
personal control B-IPQ 3	6.0 (4–8)	7.0 (5–9)	1.0 (0.6 to 1.5)	<0.0001
treatment control B-IPQ 4	7.0 (5–9)	5.0 (2–7.5)	−2.0 (−2.4 to −1.6)	<0.0001
identity B-IPQ 5	5.0 (3–7)	3.0 (2–5)	−2.0 (−2.3 to −1.7)	<0.0001
concern B-IPQ 6	5.0 (3–7)	3.0 (2–5)	−2.0 (−2.4 to −1.6)	<0.0001
coherence B-IPQ 7	8.0 (5–9)	8.0 (5–9)	0	0.275
emotional representation B-IPQ 8	2.0 (1–5)	2.0 (1–2)	0	<0.0001

Values are median (IQR) unless otherwise stated. * Statistical significance accepted at *p* < 0.05. ^¶^ Corresponding to NICE framework for self-reported knee OA. KOOS: Knee Injury and Osteoarthritis Outcome Score, higher scores denote higher functional level. Brief-IPQ (B-IPQ): Brief Illness Perception Questionnaire, higher scores on items 1, 2, 5, 6, and 8 denote a more threatening view of the illness, while higher scores on item 3, 4, and 7 denote a less threatening view of the illness. EQ5D: EuroQol-5 Domain, higher scores denote better quality of life.

**Table 2:** Health outcome scores between clusters of knee pain perception.

	Cluster 1 “Concerned Optimists” (*n* = 642)	Cluster 2 “Unconcerned Confident” (*n* = 910)	Difference	
	Median (IQR)	Median (IQR)	Median (95% CI)	*p* *
**Demographics**				
Women (N, %)	416 (64.8)	577 (63.4)	N/A	0.57
Age (median, IQR)	64 (62–67)	64 (62–67)	N/A	0.476
BMI (median, IQR)	26.8 (23.8–30.7)	25.2 (22.8–28.36)	1.6 (1.1 to 2.1)	<0.0001
Knee OA ^¶^	321 (50)	178 (19.6)	N/A	<0.0001
**KOOS. 0–100 score (median, IQR)**				
KOOS Symptoms	53.6 (46.4–60.7)	64.3 (57.1–67.9)	−10.7 (−12.1 to −9.3)	<0.0001
KOOS Qol	50 (37.5–56.2)	75 (62.5–81.2)	−25 (−25.7 to −24.3)	<0.0001
KOOS Pain	66.7 (55.6–77.8)	86.1 (80.6–91.7)	−19.4 (−20.0 to −18.8)	<0.0001
KOOS Function	72.1 (57.4–83,8)	92.6 (85.30–97.1)	−20.5 (−21.3 to −19.8)	<0.0001
KOOS Sports and recreation	35 (20–51.25)	70 (55–85)	−35 (−38 to −32)	<0.0001
**EQ5D Index, median (IQR)**	0.756 (0.723–0.824)	0.824 (0.818–1.000)	−0.068 (−0.074 to −0.062)	<0.0001
**Current VAS Pain, median (IQR)**	40 (20–62)	11 (3–24)	29 (26.4 to 31.6)	<0.0001
**User types median (IQR)**				
Non-users (N, %)	161 (25.1)	468 (51.4)	N/A	<0.0001
(only) CAM users	106 (10.6)	167 (18.4)	N/A	<0.0001
(only) Pharmacological treatment users ^#^	28 (4.4)	10 (1.1)	N/A	
(only)Non-pharmacological treatment users ^¤^	90 (14)	131 (14.4)	N/A	
Two or more treatment types ^§^	257 (40)	134 (14.7)	N/A	
**Brief-IPQ, 1–10 score (median, IQR)**				
consequences B-IPQ 1	5 (4–7)	2 (2–3)	3 (2.9 to 3.1)	<0.0001
timeline B-IPQ 2	10 (8–10)	8 (4–10)	2 (1.7 to 2.3)	<0.0001
personal control B-IPQ 3	5 (3–7)	8 (5–9)	−3 (−3.4 to −2.6)	<0.0001
treatment control B-IPQ 4	8 (6–10)	5 (2–7)	3 (2.7 to 3.3)	<0.0001
identity B-IPQ 5	6 (5–7)	3 (2–4)	3 (2.9 to 3.1)	<0.0001
concern B-IPQ 6	7 (5–8)	2 (2–3)	5 (4.7 to 5.3)	<0.0001
coherence B-IPQ 7	7 (5–9)	8 (5–9)	−1 (−2.8 to 0.8)	0.275
emotional representation B-IPQ 8	4 (2–6)	1 (1–2)	3 (2.9 to 3.1)	<0.0001

Values are median (IQR), unless otherwise stated. * Statistical significance accepted at *p* < 0.05. ^¶^ Corresponding to NICE framework for self-reported knee OA. KOOS: Knee Injury and Osteoarthritis Outcome Score, higher scores denote higher functional level. Brief-IPQ (B-IPQ): Brief Illness Perception Questionnaire, higher scores on items 1, 2, 5, 6, and 8 denote a more threatening view of the illness, while higher scores on item 3, 4, and 7 denote a less threatening view of the illness. EQ5D: EuroQol-5 Domain, higher scores denote better quality of life. CAM: Conventional and alternative medicine. ^#^ Use of over the counter medication and prescription drugs. ^¤^ Use of physiotherapy, chiropractic, or weight loss. ^§^ Use of two or more treatment types (CAMS, non-pharmacological or pharmacological treatments).

## Data Availability

The data presented in this study are available on request from the corresponding author. The data are not publicly available due to Danish and European law.

## Supplementary Materials

The following are available online at https://www.mdpi.com/2077-0383/10/4/668/s1: Supplementary file 1: Predefined choices, Supplementary file 2: Prevalence of knee OA.
