# Supplementary material for: Estimating the Prevalence of Knee Pain and the Association between Illness Perception Profiles and Self-Management Strategies in the Frederiksberg Cohort of Elderly Individuals with Knee Pain: A Cross-Sectional Study
Source: J Clin Med. 2021 Feb 9;10(4):668. doi: 10.3390/jcm10040668 (PMC7916203; doi:10.3390/jcm10040668)
Supplement: Supplementary file 1 [file jcm-10-00668-s001.zip › Supplementary file 1 Predefined choices.pdf]

Predefined choices concerning use of complementary and alternative medicine

Do you take any of the following vitamins or minerals, daily or almost daily SPECIFICALLY for your knee pain/discomfort?<br>(Please tick the appropriate boxes)	☐ no, I do not take vitamins or minerals daily or almost daily for my knee pain/discomfort<br>☐ vitamin A<br>☐ vitamin B1<br>☐ vitamin B12<br>☐ vitamin C<br>☐ vitamin D<br>☐ vitamin D with calcium<br>☐ vitamin E<br>☐ calcium<br>☐ vitamin K<br>☐ multivitamin<br>☐ zinc<br>☐ iron<br>☐ chromium<br>☐ selenium<br>☐ magnesium<br>☐ potassium<br>☐ iodine<br>☐ other
Do you take any of the following dietary supplements or herbal medicines daily or almost daily SPECIFICALLY for your knee pain/discomfort?<br>(Please tick the appropriate boxes)	☐ no, I do not take dietary supplements/herbal medicines daily or almost daily for my knee pain/discomfort<br>☐ rosehip<br>☐ ginger<br>☐ ginkgo biloba<br>☐ fish oil/cod liver oil<br>☐ garlic<br>☐ buckthorn<br>☐ yeast<br>☐ turmeric<br>☐ aloe vera<br>☐ avocado soybean<br>☐ seaweed<br>☐ flaxseed oil<br>☐ echinacea<br>☐ revadol<br>☐ ginseng<br>☐ glucosamine<br>☐ medical cannabinoids<br>☐ litomove<br>☐ vitae pro<br>☐ zincuflex<br>☐ longovital<br>☐ chlorella<br>☐ probiotic<br>☐ scanalka<br>☐ chondroitin<br>☐ other

Have you, within the last year, received any of the following non-medical treatments, SPECIFICALLY for your knee pain/discomfort?
(Please tick the appropriate boxes)

- ☐ no, I have not received any non-medical treatments for my knee pain/discomfort
- ☐ acupuncture
- ☐ hormone therapy
- ☐ acupressure
- ☐ hypnosis
- ☐ massage
- ☐ body-sds
- ☐ psychotherapeutic treatments
- ☐ healing
- ☐ reflexology
- ☐ homeopathy
- ☐ numerology
- ☐ biopathy/naturopathi
- ☐ cranio sacral therapy
- ☐ kinesiology
- ☐ iridology
- ☐ herbal therapy/phytotherapy
- ☐ other
