# Supplementary material for: Estimating the Prevalence of Knee Pain and the Association between Illness Perception Profiles and Self-Management Strategies in the Frederiksberg Cohort of Elderly Individuals with Knee Pain: A Cross-Sectional Study
Source: J Clin Med. 2021 Feb 9;10(4):668. doi: 10.3390/jcm10040668 (PMC7916203; doi:10.3390/jcm10040668)
Supplement: Supplementary file 1 [file jcm-10-00668-s001.zip › Supplementary file 2 Prevalence of Knee OA.pdf]

Estimating the Prevalence of Knee OA in the Frederiksberg Cohort (at baseline)

In probability theory and statistics, Bayes' theorem, describes the probability of an event, based on prior knowledge of conditions that might be related to the event. For example, if the risk of having knee OA (i.e. NICE definition) based on a sample of individuals with self-reported knee pain, Bayes' theorem allows the risk to be assessed more accurately (by conditioning it on knee pain) than simply assuming that the individual is typical of the population as a whole.

When applied, the probabilities involved in the theorem may have different probability interpretations. With Bayesian probability interpretation, the theorem expresses how a degree of belief, expressed as a probability, should rationally change to account for the availability of related evidence. Bayesian inference is fundamental to Bayesian statistics:

$$P(A|B) = \{P(B|A)*P(A)\} / P(B)$$

Where A and B are events and $P(B) \neq 0$.

This correspond to:

$$P(OA|KP) = \{P(KP|OA)*P(OA)\} / P(KP)$$

Where OA and KP are Osteoarthritis and Knee Pain, respectively.

This also correspond to:

$$P(KP|OA)*P(OA) = P(OA|KP)*P(KP)$$

$$P(OA) = \{P(OA|KP)*P(KP)\} / P(KP|OA)$$

From our survey sample we have the following estimates available

$P(OA|KP)$ = 32.4% (=570/1758)

$P(KP)$ = 21.4% (=1758/8204)

$P(KP|OA)$ = 100.0% (per definition, according to the sampling technique).

Thus:

$$P(OA) = (0.324*0.214)/1.000 = 6.9\%$$

From our bootstrap resampling technique, we have an empirical interval around the proportion having self-reported knee pain ranging from 19.9% to 22.7%. From this sample it is fair to assume that the prevalence of knee OA is between 6.4% and 7.4% in this age group of individuals in the age 60 to 69 years of age.

However, since this is probably a low-level *guestimate* we also applied a conservative multiple imputation technique replacing the missing data (i.e. based on a tipping point analysis strategy); from these repeated datasets (combined using Rubin's rule) a conservative estimate on individuals with self-reported knee pain could be as high as 54.2% (rather than 21.4%). As a consequence, it is possible that the prevalence of knee OA in this sample might be as high as 17.6%.
